# Combined use of hydration and alprostadil for preventing contrast-induced nephropathy following percutaneous coronary intervention in elderly patients

**DOI:** 10.3892/etm.2013.1258

**Published:** 2013-08-08

**Authors:** RONG-HE XU, GUI-ZHOU MA, ZHI-XIONG CAI, PING CHEN, ZHI-DAN ZHU, WEN-LIANG WANG

**Affiliations:** Department of Cardiology, Affiliated Shantou Hospital of Sun Yat-sen University, Shantou, Guangdong 515031, P.R. China

**Keywords:** alprostadil, percutaneous coronary intervention, contrast-induced nephropathy, elderly patient

## Abstract

Contrast-induced nephropathy (CIN) is a complex syndrome of acute kidney injury that follows exposure to intravascular contrast media. Although a series of preventive measures have been developed, CIN remains a major challenge encountered in elderly patients by interventional cardiologists. No data are currently available concerning the potential effects of the combined use of hydration and alprostadil in the prevention of CIN following percutaneous coronary intervention (PCI) in elderly patients. Therefore, the aim of the present study was to investigate the ability of a combination of hydration and alprostadil to prevent CIN following PCI in elderly patients. From June 1, 2010 to January 31, 2012, 85 elderly patients undergoing PCI were included in the present study. The included patients were randomly allocated into three groups: the control (22 cases), hydration (28 cases) and hydration + alprostadil (35 cases) group. Serum creatinine (SCr) levels were measured prior to PCI and then daily for 3 days following PCI. Creatinine clearance (Ccr) was also calculated. Following investigation of the incidence of CIN, a significant decline in Ccr was observed in the control group but not in the hydration + alprostadil group after PCI. The reduction in the level of Ccr from baseline in the hydration + alprostadil group was the smallest among the three groups. Moreover, the highest incidence of CIN was in the control group (6 cases, 27.27%), followed by the hydration group (3 cases, 10.71%) and the hydration + alprostadil group (1 case, 2.86%). Therefore, the combined use of hydration and alprostadil significantly reduces the incidence of CIN in elderly patients undergoing PCI. Hydration and alprostadil are suggested to act synergistically to protect renal function. In conclusion, the combined use of hydration and alprostadil is more effective in the prevention of CIN in elderly patients undergoing PCI compared with hydration alone.

## Introduction

Contrast-induced nephropathy (CIN) is a complex syndrome of acute kidney injury that follows exposure to intravascular contrast media. CIN is the third leading cause of hospital-acquired renal failure (1) and is associated with poor outcomes, particularly in patients undergoing primary percutaneous coronary intervention (PCI) (2–4). Numerous risk factors for CIN have been identified, including preexisting impairment of renal function, diabetes mellitus with associated renal insufficiency, nephrotoxic drugs, reduction of effective intravascular volume, multiple myeloma, volume and timing of contrast administration and advancing age (5). These risk factors may be additive (5). Due to the age-associated degradation in physiological functions and a higher prevalence of chronic diseases, elderly patients have more risk factors. Therefore, older age was shown to be a strong risk factor for CIN (6). Although a series of preventive measures have been developed, CIN remains a major challenge encountered in elderly patients by interventional cardiologists.

Hydration is generally regarded as an important strategy for preventing CIN. However, the optimal therapy for preventing CIN remains uncertain (7). Prostaglandin E_1_ (PGE_1_), a natural prostaglandin with numerous pharmacologic effects, has been reported to have potential as an agent for preventing CIN (8–9). However, no data are currently available concerning the potential effects of the combined use of hydration and alprostadil in the prevention of CIN following PCI in elderly patients. Therefore, the aim of the present study was to investigate the ability of a combination of hydration and alprostadil to prevent CIN following PCI in elderly patients.

## Patients and methods

### Patient population

The present study included elderly patients (aged ≥60 years) with coronary artery disease who were admitted to the Affiliated Shantou Hospital of Sun Yat-sen University (Shantou, China) between June 1, 2010 and January 31, 2012, and treated with PCI. Patients were excluded from the present study when the following criteria were met: i) refusal to participate in the clinical trial, ii) refusal of PCI treatment, iii) use of any nephrotoxic drugs during the perioperative period, iv) severe hepatic and renal failure, v) serious infectious disease, vi) New York Heart Association Functional Classification (NYHA) (10) class >3, vii) hemodynamic instability (including systolic blood pressure <90 mmHg), viii) coronary lesions below the threshold for clinical revascularization therapy, ix) coronary lesions not suitable for PCI due to coronary anatomy, and x) allergic reaction to contrast media and alprostadil. The present study was approved by the Ethics Committee of the Affiliated Shantou Hospital of Sun Yat-sen University, and informed consent was obtained from all the patients.

### Treatment regimens

According to the American Heart Association (AHA)/American College of Cardiology (ACC) guidelines for secondary prevention for patients with coronary artery disease (11), all the patients following admission were treated with antiplatelet and other therapies, including angiotensin-converting enzyme inhibitors/angiotensin II receptor blockers (ARBs), β-blockers and statins.

All the patients underwent coronary angiography and percutaneous transluminal coronary angioplasty and/or implantation of stents according to the condition of the coronary artery. According to interventional guidelines of coronary artery disease (ACC/AHA 2005) (12), >70% narrowing of local stenosis of the culprit lesions (lesions directly responsible for the ischemia episode) is the threshold for clinical revascularization therapy (13). Iopromide was the non-ionic, monomeric, hypo-osmolar contrast media employed during PCI.

Following admission, the patients were randomly allocated to one of three groups: i) the control group, where treatment was based on the routine PCI without hydration; ii) the hydration group, where routine hydration was performed with 1 ml/kg/h normal saline for 6 h prior to PCI and 12 h following PCI; and iii) the hydration + alprostadil group, where, on the basis of hydration, patients received alprostadil 10 *μ*g (diluted with 100 ml normal saline) twice a day by intravenous drip for the 3 days following PCI.

### Clinical and laboratory monitoring

The serum creatinine (SCr) concentration was measured prior to PCI and then daily for three days following PCI. The serum lipid profile and fasting blood glucose (FBG) were also measured during hospitalization. Transthoracic echocardiography was performed prior to PCI to evaluate the left ventricular ejection fraction (LVEF) in all the patients, and the results were recorded and interpreted by experienced experts.

### Definitions

CIN was defined as a relative increase of >25% or an absolute increase of ≥0.5 mg/dl in SCr from the baseline value 72 h after exposure to the contrast medium (14). Creatinine clearance (Ccr) was calculated by applying the Cockcroft-Gault formula to the SCr (15): Ccr = (140 – age) × weight (kg)/[SCr (mg/dl) × 72], with adjustment for female gender (Ccr_female_ = Ccr × 0.85).

### Statistical analysis

Statistical analysis was performed using SPSS version 16.0 (SPSS, Inc., Chicago. IL, USA). Continuous variables following a symmetric distribution were presented as means ± standanrd deviation (SD). Comparisons between means were performed using Student’s t-test, while comparisons between frequencies were performed using Chi-square or Fisher’s exact tests. Variables following an asymmetric distribution were presented as medians and interquartile ranges [M (P25, P75)] and were compared using the Kruskal-Wallis nonparametric test. Categorical variables were described as frequencies and analyzed by Chi-square or Fischer’s exact tests. P≤0.05 was considered to indicate a statistically significant difference.

## Results

### Patient characteristics

From June 1, 2010 to January 31, 2012, 85 elderly patients were included in the present study after exclusions. All the included patients completed the full clinical trial. The patients were randomly allocated to the control, hydration and hydration + alprostadil groups. In the hydration + alprostadil group, two patients were observed to have mild drug-related phlebitis. Moreover, no cardiovascular hypotensive side-effects were observed. The baseline clinical, laboratory and procedural characteristics of the study population are shown in Table I.

### SCr

SCr values (mean ± SD) measured at the four time points specified in this study are shown in Table II. The mean SCr of the three groups increased gradually during the three days following PCI. The highest SCr value was observed in the control group. However, in the remaining two groups (hydration and hydration + alprostadil groups), the SCr values were only mildly increased. The mean SCr values at baseline did not differ significantly following analysis of variance (P=0.277). However, Kruskal-Wallis test comparisons of SCr values on the 3rd day after PCI demonstrated a significant (Chi square=10.527, P=0.005) difference among the three groups.

### Ccr

**Figure 1.**
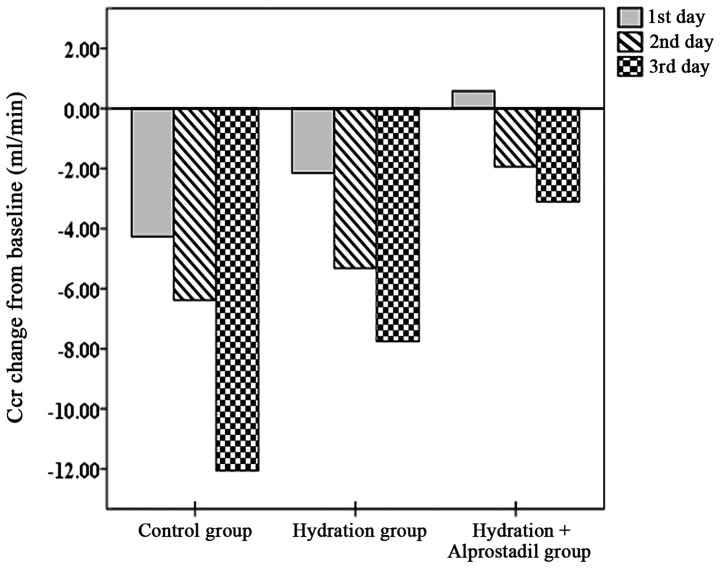
Mean change in Ccr value from baseline following PCI. ① Control group; ① hydration group; ③ hydration + alprostadil group. Day 1 after PCI: ① vs. ② and ② vs. ③, P>0.05; ① vs. ③, P<0.05. Day 2 after PCI: ① vs. ② and ② vs. ③, P>0.05; ① vs. ③, P<0.05. Day 3 after PCI: ① vs. ②, P<0.05; ① vs. ③ and ② vs. ③, P<0.01. Ccr, creatinine clearance; PCI, percutaneous coronary intervention.

The mean changes in Ccr from baseline for the three groups are shown in Fig. 1. On the first day after PCI, the Ccr in the control group had decreased moderately. The reduction in the Ccr was less evident in the remaining two groups. However, no statistically significant difference was observed in the mean changes of Ccr between the control and hydration groups. A statistically significant difference was observed between the control and hydration + alprostadil groups. On the second day after PCI, the mean changes in Ccr from baseline for the three groups were similar to those on the first day after PCI. No statistically significant difference was observed in the mean changes of Ccr between the control and hydration groups. However, the difference was statistically significant between the control and hydration + alprostadil groups. On the third day after PCI, the Ccr values among the three groups were statistically significant from baseline. Notably, the lowest reduction in the Ccr value was observed in the hydration + alprostadil group, while the highest reduction was observed in the control group. The Ccr value in the hydration group was decreased to a lesser extent compared with that in the control group (P<0.05). A smaller reduction in the Ccr value was observed in the hydration + alprostadil group than in the hydration group (P<0.01).

### Incidence of CIN

In the present study, 10 patients developed CIN following PCI and the overall incidence reached 11.76%. There were six patients in the control group, three in the hydration group and one in the hydration + alprostadil group that developed CIN. The incidence of CIN in the three groups was 27.27, 10.71 and 2.86%, respectively. The Kruskal-Wallis test comparisons of the incidence of CIN demonstrated a significant difference among the three groups (Chi square=7.802, P=0.05).

## Discussion

To the best of our knowledge, this is the first study to provide information regarding the potential effects of the combined use of hydration and alprostadil in the prevention of CIN following PCI in elderly patients. Our findings demonstrate that the combined use of hydration and alprostadil is more effective in preventing CIN in elderly patients undergoing PCI compared with hydration alone.

Older age is an important risk factor for CIN since elderly people often suffer from age-related diseases (6). These diseases contribute to an increased incidence of CIN. Therefore, it is important to prevent CIN in this population. Many preventive strategies for reducing the incidence of CIN have been reported. However, such strategies, except for intravenous hydration, have yielded disappointing outcomes (1,16). Previous studies (8,9) have shown that the intravenous administration of PGE_1_ is beneficial in reducing the incidence of CIN. The main mechanism is considered to relate to a reduction in the levels of prostaglandins when CIN develops, which causes a shift in the physiologic vasoconstriction/vasodilatation balance. Alprostadil, an exogenous form of PGE_1_, is suggested to cause renal vasodilation and active renal artery perfusion, and to counteract contrast-induced renal tubular epithelial cell toxicity (9,17).

In the present study, the effectiveness of hydration combined with alprostadil in the prevention of CIN in elderly patients undergoing PCI was investigated. The Ccr values in the hydration group were not significantly different in the first and second day after PCI compared with those in the control group. However, on the third day after PCI, the Ccr in the control group decreased more markedly compared with that in the hydration group. The incidence of CIN in the control and hydration groups was 27.27 and 10.71%, respectively. This finding demonstrated that hydration was effective in reducing the incidence of CIN. However, the incidence of CIN was still higher compared with that in the general population (18). Therefore, hydration alone was not sufficient in preventing CIN in elderly patients undergoing PCI.

In the present study, the combined use of hydration and alprostadil showed an evident protective effect on renal function from the first day after PCI. The influence of the contrast medium on Ccr in the hydration + alprostadil group was limited. However, the Ccr levels in the control and hydration groups were differentially reduced, particularly in the control group. A clear advantage was observed following the combined use of hydration and alprostadil after the third day following PCI, when compared with the hydration group. Finally, the incidence of CIN in the hydration and hydration + alprostadil groups was 10.71 and 2.86%, respectively. Our findings indicate that the combined use of hydration and alprostadil is more effective in reducing the incidence of CIN. Therefore, hydration and alprostadil are suggested to act synergistically to protect renal function.

Studies by Koch *et al* (8) and Sketch *et al* (9) concerning the duration and dosage of intravenously administered PGE_1_ obtained similar results. In these studies, PGE_1_ administration was implemented 1 h prior to contrast exposure and was continued for a total of 6 h. The time interval was chosen based on the knowledge of the half-life of contrast media excretion (19). The most effective dosage used was 20 ng/kg/min (8,9). However, concerning the clinical characteristics of CIN, according to the literature, SCr usually increases 24 h after contrast exposure and peaks within 48–72 h (14,20). In the present study, based on the above-mentioned clinical characteristics of CIN, alprostadil was administered by intravenous drip for the 3 days following PCI. Finally, our study confirmed the clear effect of the combined use of hydration and alprostadil in reducing the incidence of CIN in elderly patients undergoing PCI.

Alprostadil is known to lower blood pressure via vasodilation. Therefore, alprostadil is suggested to aggravate contrast media nephrotoxicity by a severe drop in blood pressure. According to the study by Koch *et al* (8), there was no severe drop in blood pressure when the protective effect of PGE_1_ reached a peak at a dosage of 20 ng/kg/min. In the present study, patients were treated with 10 *μ*g alprostadil (diluted with 100 ml normal saline) twice a day by intravenous drip for the 3 days following PCI. No severe drop in blood pressure was observed, with the exception of mild drug-related phlebitis. This result may be attributed to the following factors: i) the dose of alprostadil used was relatively low; and ii) hydration may increase the blood volume and help prevent hypotension. Therefore, the findings of our study also suggest that alprostadil, when combined with hydration, protects renal function more effectively.

In conclusion, the present study showed that the combined use of hydration and alprostadil significantly reduce the incidence of CIN in elderly patients undergoing PCI. Hydration and alprostadil are suggested to act synergistically to protect renal function. The combined use of hydration and alprostadil is more effective in the prevention of CIN in elderly patients undergoing PCI compared with hydration alone.

However, the present study has several limitations. Firstly, the number of each group of patients was limited. Secondly, data were collected from a single center, and, finally, this was not a double-blind study. Therefore, the results provided by this study should be confirmed by a larger double-blind multi-center study.

## Figures and Tables

**Table I.** Baseline clinical, laboratory and procedural characteristics of the patients included in the present study.

Characteristic	Group			P-value
	Control (n=22)	Hydration (n=28)	Hydration + alprostadil (n=35)	
Mean age (years)	71±8	69±6	70±7	0.540
Male, n	15	19	26	0.822
Scr (mol/l)	88.27±27.40	76.82±19.45	83.63±23.59	0.277
Ccr (ml/min)	63.59±20.07	66.54±18.29	64.07±20.38	0.759
LDL-C (mmol/l)	2.95±0.96	2.93±0.97	2.94±1.08	0.997
FBG (mmol/l)	6.61±2.68	7.40±3.04	6.75±1.67	0.424
LVEF ≤ 45%, n (%)	4 (18.18)	5 (17.86)	6 (17.14)	0.994
Past history				
Hypertension, n (%)	12 (54.55)	13 (46.43)	15 (42.86)	0.688
Diabetes mellitus, n (%)	7 (31.82)	13 (46.43)	14 (40.00)	0.578
Renal insufficiency, n (%)	1 (4.55)	3 (10.71)	6 (17.14)	0.348
Combined medications				
ACEIs/ARBs, n (%)	20 (90.91)	25 (89.29)	29 (82.86)	0.618
Diuretic agents, n (%)	2 (9.09)	5 (17.86)	5 (14.29)	0.676
Mean contrast volume (ml)	134.09±36.99	123.57±37.14	133.71±32.46	0.400

Values for SCr, Ccr, LDL-C, FBG and mean contrast volume are mean ± standard deviation. SCr, serum creatinine; Ccr, creatinine clearance; LDL-C, low-density lipoprotein cholesterol; FBG, fasting blood glucose; LVEF, left ventricular ejection fraction; ACEIs, angiotensin-converting enzyme inhibitors; ARBs, angiotensin II receptor blockers.

**Table II.** Time course of serum creatinine (SCr) (mean ± SD, *μ*mol/l).

Time point	Group		
	Control	Hydration	Hydration + alprostadil
Baseline	88.27±27.4	76.82±19.45	83.63±23.59
1st day after PCI	95.63±31.21	79.79±23.38	83.47±26.46
2nd day after PCI	99.23±34.20	83.41±21.80	85.43±23.70
3rd day after PCI	107.48±30.45	87.11±22.72	86.49±21.86

PCI, percutaneous coronary intervention.
